# Usability Testing of the Kidney Score Platform to Enhance Communication About Kidney Disease in Primary Care Settings: Qualitative Think-Aloud Study

**DOI:** 10.2196/40001

**Published:** 2022-09-28

**Authors:** Delphine S Tuot, Susan T Crowley, Lois A Katz, Joseph Leung, Delly K Alcantara-Cadillo, Christopher Ruser, Elizabeth Talbot-Montgomery, Joseph A Vassalotti

**Affiliations:** 1 University of California San Francisco San Francisco, CA United States; 2 Veterans Affairs Connecticut Healthcare System West Haven, CT United States; 3 Veterans Affairs New York Harbor Healthcare System New York, NY United States; 4 Yale University New Haven, CT United States; 5 National Kidney Foundation New York, NY United States; 6 Icahn School of Medicine at Mount Sinai New York, NY United States

**Keywords:** chronic kidney disease, CKD, awareness, usability, kidney, renal, think aloud, self-management, patient education, health education

## Abstract

**Background:**

Patient awareness of chronic kidney disease (CKD) is low in part due to suboptimal testing for CKD among those at risk and lack of discussions about kidney disease between patients and clinicians. To bridge these gaps, the National Kidney Foundation developed the Kidney Score Platform, which is a web-based series of tools that includes resources for health care professionals as well as an interactive, dynamic patient-facing component that includes a brief questionnaire about risk factors for kidney disease, individualized assessment of risk for developing CKD, and self-management tools to manage one’s kidney disease.

**Objective:**

The aim of this study is to perform usability testing of the patient component of the Kidney Score platform among veterans with and at risk for kidney disease and among clinicians working as primary care providers in Veterans Affairs administration.

**Methods:**

Think-aloud exercises were conducted, during which participants (veterans and clinicians) engaged with the platform while verbalizing their thoughts and making their perceptions, reasonings, and decision points explicit. A usability facilitator observed participants’ behaviors and probed selectively to clarify their comprehension of the tool’s instructions, content, and overall functionality. Thematic analysis on the audio-recording transcripts was performed, focusing on positive attributes, negative comments, and areas that required facilitator involvement.

**Results:**

Veterans (N=18) were 78% (14/18) male with a mean age of 58.1 years. Two-thirds (12/18) were of non-White race/ethnicity, 28% (5/18) had laboratory evidence of CKD without a formal diagnosis, and 50% (9/18) carried a diagnosis of hypertension or diabetes. Clinicians (N=19) were 29% (5/17) male, 30% (5/17) of non-White race/ethnicity, and had a mean of 17 (range 4-32) years of experience. Veterans and clinicians easily navigated the online tool and appreciated the personalized results page as well as the inclusion of infographics to deliver key educational messages. Three major themes related to content and communication about risk for CKD emerged from the think-aloud exercises: (1) tension between lay and medical terminology when discussing kidney disease and diagnostic tests, (2) importance of linking general information to concrete self-management actions, and (3) usefulness of the tool as an adjunct to the office visit to prepare for patient-clinician communication. Importantly, these themes were consistent among interviews involving both veterans and clinicians.

**Conclusions:**

Veterans and clinicians both thought that the Kidney Score Platform would successfully promote communication and discussion about kidney disease in primary care settings. Tension between using medical terminology that is used regularly by clinicians versus lay terminology to promote CKD awareness was a key challenge, and knowledge of this can inform the development of future CKD educational materials.

## Introduction

Chronic kidney disease (CKD) is a chronic disease that requires individual participation in health-related behaviors to decrease the risk of progression and associated cardiovascular disease [1]. Patient awareness of CKD—including the knowledge of having a kidney problem, the perceived risk of developing kidney disease, and ability to affect one’s kidney health—is necessary for patients to participate in shared decision-making about their kidney health and to apply management recommendations to improve outcomes [2]. However, as many as half of patients with advanced CKD are unaware that they have kidney disease, including those at high risk for kidney function decline [3] and those with laboratory manifestations of their kidney disease [4,5].

Reasons for the low prevalence of CKD awareness among individuals with CKD are varied and include patient, provider, and health system factors [6]. Two of these contributing factors are suboptimal testing for CKD among those at risk for kidney disease [7,8] and lack of discussions about kidney disease between patients and clinicians among those individuals with laboratory-documented CKD. Studies in primary care settings have consistently demonstrated that discussions about kidney disease occur less frequently than do conversations about other chronic diseases [9] and that primary care clinicians experience challenges in improving their patients’ understanding of kidney disease, even when using principles of shared decision-making [10,11]. Additionally, individuals at risk for CKD have low perceived risk of the condition [12], which may exacerbate primary care clinicians’ concerns of emotionally overwhelming patients with a diagnosis of CKD [6].

To bridge the communication gap about kidney disease among patients and health care professionals and increase testing among individuals at risk for CKD, the National Kidney Foundation (NKF) developed the Kidney Score Platform, leveraging the behavior change wheel, a validated framework used to design interventions to incite individual behavior change [13]. The Kidney Score Platform is a web-based series of tools that includes resources for health care professionals to encourage the use of a population health strategy for CKD management [14] as well as an interactive, dynamic component for patients to increase their knowledge and self-management. Before embarking on a study of the Kidney Score Platform’s impact on patient-clinician communication about kidney disease and individual awareness of CKD [13], we sought feedback from clinicians about the provider resources available on the Kidney Score Platform and engaged in usability testing of the patient-facing tool to gather feedback regarding its acceptability and potential use [15]. A partnership between the NKF and Veterans Administration provided an opportunity to perform usability testing of the patient component of the Kidney Score Platform among veterans with and at risk for kidney disease and among clinicians working as primary care providers in Veterans Affairs administration. Here, we describe our experience with that usability testing, which culminated in important refinements to the Kidney Score Platform, which is now ready for an examination of its impact on patient-clinician conversations about kidney disease in Veterans Administration ambulatory settings.

## Methods

### Patient Education Tool

**Figure 1:**
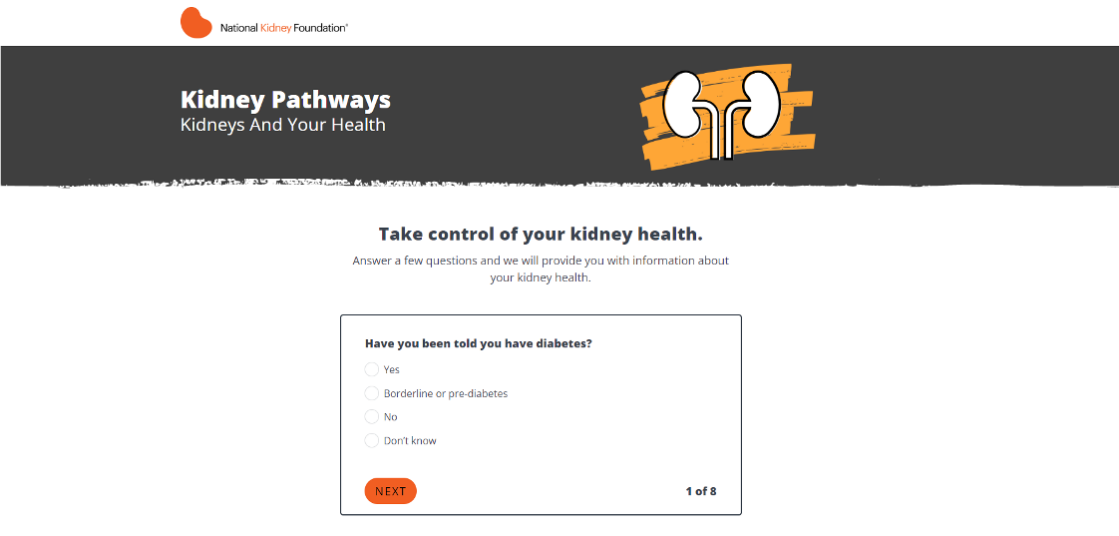
Example questions within the CKD risk self-assessment tool. CKD: chronic kidney disease.

**Figure 2:**
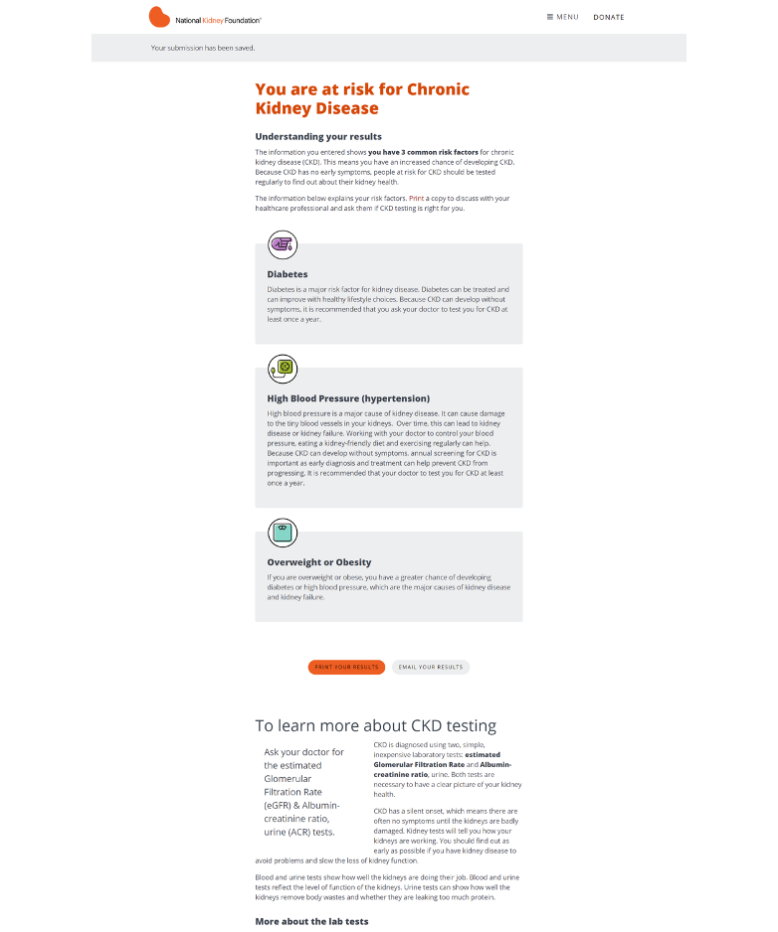
Example self-assessment results, linking risk factors to kidney disease risk, providing education about CKD diagnostic tests, and encouraging patients to review diagnostic tests with their primary care clinician to increase awareness of CKD. CKD: chronic kidney disease.

**Figure 3:**
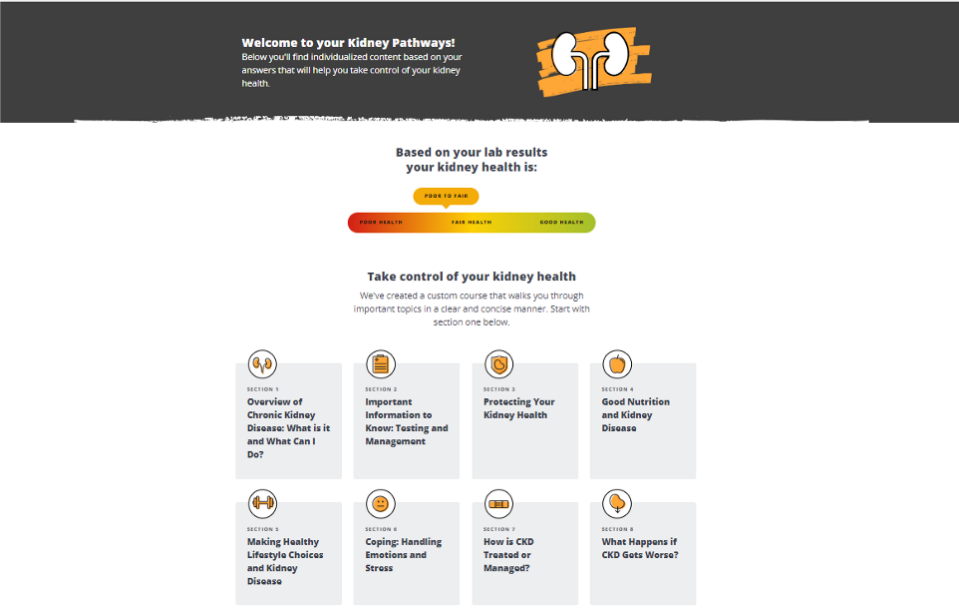
Patient educational materials to promote CKD self-management. This page is available to individuals who document that they are aware of their own kidney disease. CKD: chronic kidney disease.

The patient-facing component of the Kidney Score platform includes a brief questionnaire about risk factors for kidney disease that results in a personalized educational results page providing an individualized assessment of risk for developing CKD or self-management tools to manage one’s kidney disease (Figures 1-3). Development of the tool has been described in depth elsewhere [13].

### Participant Selection

The Kidney Score Platform was field tested in 2 phases among 20 veterans and 19 clinicians from the VA NY Harbor Healthcare System (VA-NYHHS) and the VA CT Healthcare System at West Haven (VA-CTHS). We used a purposeful sampling approach using the electronic medical record to identify potential veteran participants who were English-speaking, active primary care patients between the ages of 18 and 75 years and who were living with diabetes or hypertension, the 2 most common causes of chronic kidney disease in the United States. Although having kidney disease was not an inclusion or exclusion criterion, we excluded veterans with very advanced kidney disease, including those with an estimated glomerular filtration rate (eGFR) <15 ml/min/1.73m^2^, those receiving dialysis treatments, and individuals who were kidney transplant recipients. Veterans who were unable to use a tablet or computer device (ie, blind, illiterate, with moderate-to-severe dementia) were also excluded. Potential veteran participants were mailed a flyer about the study and provided a phone number to opt out of the study. A research coordinator subsequently called veterans who did not opt out of the research to explain study goals and procedures, obtain consent, arrange for hard signatures of necessary forms, and schedule the think-aloud interviews.

Clinician participants included physicians and nurse practitioners who were actively engaged in primary care delivery at either Veterans Administration site. Recruitment of clinicians occurred via email by members of the research team. A research coordinator then followed up with eligible clinicians who responded favorably to schedule the think-aloud interviews.

### Ethical Considerations

After providing online documentation of informed consent, each participant joined an online platform using a numeric study ID. Two usability facilitators (one expert consultant external to the research team and DST, a nephrologist) introduced themselves and provided a brief overview of the goals of the project. Participants were asked again to acknowledge informed consent that participation was voluntary, could cease at any time, and that the session would be audiotaped but their privacy safeguarded. The study was approved by the institutional review boards at VA-NYHHS (approval #1705) and VA-CTHS (approval #02290). The COVID-19 pandemic required protocol modifications with appropriate institutional review board amendments to allow remote participation of veterans and clinicians in online think-aloud sessions in contrast to the original investigation design, which included face-to-face study interactions. The COVID-19 pandemic also extended the research timeline, creating 2 similar phases of work instead of 1.

### Think-Aloud Testing Protocol

Each session began with a warm-up interview exploring participants’ experience with online resources related to health and kidney health. Veterans were asked about experiences using web-based resources to gain information about their own health; clinicians were asked about use of online resources to communicate about chronic diseases, including but not limited to kidney disease. Thereafter, all participants were provided a weblink to the Kidney Score Platform website and were asked to participate in a think-aloud exercise, during which they were asked to engage with the platform while verbalizing their thoughts and making their perceptions, reasonings, and decision points explicit. Think-aloud exercises are increasingly being used to conduct user testing of digital health interventions [15]. During the exercise, the usability facilitators observed participants’ behaviors and probed selectively to clarify their comprehension of the tool’s instructions, content, and experience. The facilitators also prompted participants to comment on the positive and negative aspects of the tool’s content, language, and overall functionality.

During phase 1 (March 2020 to April 2020), audio recordings of field tests with 10 veterans and 19 clinicians were reviewed. Thematic analysis was performed, with focus on positive attributes, negative comments, and areas that required facilitator involvement. Clinicians were also asked how they might use this tool in clinical practice. Areas of improvement in the layout or design of the tool were quickly addressed by the research team and the NKF, which developed a second iteration of the Kidney Score Platform. Examples of changes included the following: reducing white space in between educational sections, rearranging location of kidney graphics, and replacing a picture of a heart with the word “love” for clarity. The Kidney Score Platform was reviewed by an additional 10 veterans during phase 2 of field testing (February 2021 to March 2021). Results from both phases were used to finalize the tool.

### Data Analysis

No formal hypothesis testing was performed due to the qualitative study methods of usability testing. Participants’ demographic characteristics are described using counts and percentages. Notes from direct (although online) observations by the usability facilitators and audio recordings of the field tests were reviewed. Thematic analysis was performed (without any special software), with a focus on themes derived from the data regarding positive attributes, negative comments, and areas that required facilitator involvement.

## Results

### Participant Characteristics

Veterans were 78% (14/18) male with a mean age of 58.1 years (range 27-71 years); see Table 1. Over 60% (11/18) of veterans self-identified as African American and 6% (1/18) were Hispanic. Nearly one-third (5/18) had laboratory evidence of CKD without a formal diagnosis of CKD by problem list and 50% (9/18) carried a diagnosis of diabetes. Clinicians were 29% male (5/17). Approximately 18% (3/17) were African American, 12% (2/17) were Asian, and 65% (11/17) self-identified as non-Hispanic White. Most (15/17, 88%) were physicians (vs advanced practice providers) with a mean of 17 (range 4-32) years of experience.

**Table 1:** Characteristics of study participants.

Characteristics			Veterans (N=18)		Clinicians (N=17)^a^
Male gender, n (%)			14 (78)		5 (29)
Age, mean (range)			58 (27-71)		N/A^b^
**Race/ethnicity, n (%)**					
	African American	11 (61)		3 (18)	
	Hispanic	1 (6)		0	
	Asian	0 (0)		2 (12)	
	Non-Hispanic White	6 (33)		11 (65)	
**Comorbid conditions**					
	Chronic kidney disease (eGFR^c^ 30-60 ml/min/1.73m^2^) but no diagnosis	5 (28)		N/A	
	Diagnosis of diabetes	9 (50)		N/A	
	Diagnosis of hypertension	9 (50)		N/A	
**Practitioner type**					
	Nurse practitioner	N/A		2 (12)	
	Physician	N/A		15 (88)	
	Time since completing training (years), mean (range)	N/A		17 (4-32)	

^a^Data are missing for 2 clinicians who did not respond to demographic questions.

^b^N/A: not applicable.

^c^eGFR: estimated glomerular filtration rate.

### Usability Testing Results

#### Overview

All veterans and clinicians successfully navigated to the Kidney Score Platform website after being provided a link to the home page. Thereafter, they easily followed the self-assessment questionnaire portion of the Kidney Score Platform, aided by standard button labels and a simple progress indicator (eg, “3 of 8”) that provided a clear signal about survey length, which was also considered reasonable. The visual design of the personalized results page was appealing to all participants, who also appreciated the inclusion of infographics. Clinicians and veterans both recommended to reduce the overall text copy and include links to additional educational resources on the personalized educational results page.

Three major themes related to content and communication about risk for CKD emerged from the think-aloud exercises: (1) tension between lay and medical terminology when discussing kidney disease and diagnostic tests, (2) importance of linking general information to concrete self-management actions, and (3) usefulness of the tool as an adjunct to the office visit to prepare for patient-clinician communication. Importantly, these themes were consistent among interviews involving both veterans and clinicians.

#### Tension Between Lay and Medical Terminology When Discussing Kidney Disease Risk

Clinicians affirmed that phrasing of questions such as “Have you been told” or “Do you take medications for [disease name]” were likely to yield accurate responses during self-assessment rather compared to asking patients whether they have a particular disease. Providers were concerned that patients may not associate (or identify) with heart disease or high blood pressure even though they were actively taking medications to manage these conditions, especially if these chronic conditions were being well managed. However, a question asking about “Have you been told” directly relates to prior conversations about chronic diseases, regardless of management strategy or success.

Aligned with clinician feedback, veterans generally understood the phrasing “Have you been told you have [health condition]” and related questions appeared to garner accurate responses from patients regarding their health risks. Veterans correctly answered yes to being told they had high blood pressure or heart failure even if they were taking medications to manage the underlying condition. However, some individuals struggled to assess whether being at risk for diabetes meant they had prediabetes, in part because they had not heard of the term “prediabetes” before.

So my doctor never really told me I have prediabetes. He told me my A1C.

Veteran 2

Nearly all clinicians were concerned about veterans not recognizing the medical terms “heart failure” or “chronic kidney disease.” Clinicians stated that during their communications with patients, they were more likely to describe the underlying disease rather than rely on disease names, for example, noting to patients that “your kidneys are not functioning properly” rather than using the term “chronic kidney disease.” To be consistent with the lay terminology used during clinic conversations, one primary care clinician suggested the following:

I wonder if a question like, have you been told that you have chronic kidney disease, whether it should also say something like, have you been told that you have any problems with your kidneys or if your kidneys don't work completely normally or something that might not be a phrase in medical jargon.

Clinician F

#### Tension Between Lay and Medical Terminology When Discussing Kidney Health Tests

Two questions embedded in the self-assessment tool asked about eGFR and urine albumin-creatinine ratio (uACR). Clinicians doubted that any of their patients would know their eGFR or uACR.

I’m almost sure no one would know their GFR … I think only a very special person would know [their uACR] even more so than GFR.

Clinician B

Most clinicians noted that they rarely used these terms in clinical practice when discussing kidney disease with their patients. Instead, they relied on plain language descriptions (“urine test” for uACR) and simplified conceptual explanations (“kidneys functioning at 60%” to describe eGFR). Although several providers acknowledged that this was an imprecise translation, they thought that it was important to make the information more accessible to patients to promote CKD awareness.

The question that I get most often when I talk about CKD is, “What percent of my kidneys are still functioning?” Because GFR is a hard number to remember … I’ll tell people, “Listen, I think we lost 50% of your kidney function.”

Clinician G

Clinicians had a similar approach when discussing the presence of albuminuria.

When I talk to patients about having protein in their urine, I don't reference the number all that often. Though more so than with eGFR, I'll pull up the trend … But even then, I'll rarely refer to it by name.

Clinician F

As clinicians predicted, hardly any veterans were familiar with eGFR or uACR.

This is like a foreign language to me. I've never heard those words before.

Veteran 3

For most patients, the terms themselves were new; all patients answered “I don’t know” to the questions asking about levels of eGFR and uACR (Figure 2). Even among the few veterans whose doctors has spoken to them about albumin or protein in their urine, none knew the laboratory test by its clinical name, and none knew their results beyond whether they were in a normal range.

I may not know what my numbers are, but I do know what the tests are, and I do know that I've had them done before.

Veteran 10

Patients used lay terminology to describe eGFR and uACR, consistent with what was described by clinicians. Most veterans said that their primary care clinician would talk more about the significance of the laboratory results as opposed to using specific terms or values.

My doctor just said, “You don’t have protein in your urine.” I don't know the number or whether it was elevated.

Veteran 8

I know that they have done urine tests in the past, and I know protein and sugar was in my urine.

Veteran 13

#### Desire for More Explicit Linkage to Self-Management Tools

Although clinicians appreciated the personalized nature of the individualized results page about risk factors for CKD (Figure 2), they felt that there were too few actionable items that would help patients tangibly improve their health.

I don't see much here for how to manage the risk factors… There's not much here about next steps.

Clinician F

If I were a patient, I'd like links to more info about kidney-friendly eating and exercising options. I would want to have information about medications to avoid, medications that can be helpful.

Clinician C

Similarly, while most patients understood from the results page that they were at risk for kidney disease, some did not read carefully enough to fully realize the perceived-risk concept between diabetes, heart disease, and kidney disease and that management of the diabetes and heart disease would help mitigate risk of kidney disease. Of those patients who read the results more carefully, some felt empowered by the information, while others reacted with alarm to the risk of CKD. In particular, veterans emphasized the importance of providing actionable education to help motivate individuals to change their personal risks for kidney disease without paralyzing them with the idea that kidney failure requiring dialysis or transplant was inevitable.

It's kind of gloom and doom; if I hit these thresholds, things may not be working properly, or may be approaching failure. So the message I get is: if you see these numbers, you're in deep trouble.

Veteran 3

Although most veterans planned on speaking with their clinicians about the questionnaire results, some were seeking more actionable steps that they could take on their own. They yearned for more concrete recommendations that they could adopt.

It suggests exercise, but it’s not telling me the type of exercise to do.

Veteran 1

What I would be mostly interested in is what is happening, why is it happening, and what can I do to slow it down? Anything I see that's clickable that touches on those points, I would be interested in clicking on.

Veteran 11

#### Usefulness of the Tool as an Adjunct to the Office Visit

When asked whether they would use the tool in clinical practice, most clinicians viewed the tool’s primary value was for educating patients prior to their next appointment. With limited time during office visits, clinicians did not think that they could review the tool in its entirety with patients; however, they viewed the tool as one that patients could use in preparation for an office visit that could be dedicated to a discussion about kidney disease and cardiovascular risk. Clinicians felt that patients would benefit from having time to go through the self-assessment and results on their own time, particularly if there were more actionable next steps identified for them.

If they can sort of generate the results and then bring them in, I think that would be helpful to have a discussion about where they are with their CKD and how we can help sort of reduce their risk of progression.

Clinician A

From my perspective, I wouldn’t use our visit time to go through this. Though maybe patients might find value in going through ahead of time.

Clinician H

Overall, most veterans felt the tool was useful since it made them aware of kidney disease. All participants said that they planned to email their CKD risk results to themselves to prepare for a discussion about kidney health with their clinician at their next visit.

Actually, I like the site better than I thought I would. … those questions were very precise and specific questions. As long as everybody's being honest when they're answering, I think the information that they're going to receive is going to be very useful.

Veteran 10

There's some good information here. It sounds like it at least presents [information] to you a way to have this discussion with your doctor, and then see if they can test your blood or test your urine, and do the necessary tests to see where you are.

Veteran 5

Although no patients knew their kidney-health lab values, most were intrigued enough that they planned to bring it up with their primary care clinician at their next visit.

It made me more interested in getting a test to see where I’m at.

Veteran 4

For many, being asked about their kidney health values and not knowing the answer motivated them to speak to their clinician about CKD.

Well, let me put this way: I'm now well aware now of the significance of the kidneys and about what the issues are here. And I would definitely consider... When I go to the doctor, I would say to him, “Now, listen. You did the blood tests. But how are my kidneys doing? What are the numbers?”

Veteran 6

Part of me is kind of mad there, because this a blind spot that me and my doctor, who I feel pretty comfortable with; we have not talked about CKD. I don't know if he didn't want to scare me, or maybe because he's concentrated on the prediabetes. I feel like I'm going to harass him about CKD now.

Veteran 7

This is something new, so immediately I was like, just another thing to be concerned about. But then I felt kind of empowered, and like I really do want to get ahead of this thing. I feel like I do want to have a conversation with my primary care physician. CKD makes me feel better than end stage renal failure, so that makes me feel empowered because I'm at risk for chronic kidney disease, which is not end stage renal failure.

Veteran 7

## Discussion

### Principal Findings and Comparison With Prior Work

The Kidney Score Platform is an online educational tool that was developed to promote communication and discussion about kidney disease among patients and their primary care clinicians. Usability findings demonstrate that this goal was met, with the majority of patients finding the digital tool to be helpful and easy to navigate. Content areas that would benefit from refinement were also clearly identified.

The think-aloud exercises identified 2 key themes pertinent to the development of all educational materials related to kidney disease. First, there is a tension between using medical terminology that is used regularly by clinicians as well as reinforced in laboratory reports and electronic records versus lay terminology to educate patients and promote CKD awareness. This has been an area of debate in the nephrology field for quite some time [16]. Prevalence of CKD awareness among patients differs when asked with different terms (eg, “kidney problem,” “chronic kidney disease,” “weak or failing kidneys”) [17-19]. Weak or failing kidneys is currently used in the most widely cited metric for CKD awareness in the United States, implying that low awareness is at least in part the result of semantics. Use of lay terms to describe CKD (“kidney problem”), eGFR (“percentage of kidneys filtering well”), and uACR (“protein in the urine”) in educational tools may reinforce communication about kidney disease that occurs during clinical encounters.

The use of low grade-level vocabulary is an important component of adult education and written education materials [20]. However, lay terminology is nonspecific and poses challenges for clinicians to describe the pathophysiology underlying kidney disease and to share with patients how behavioral interventions or medications will help slow kidney disease decline [21]. Complicating matters, eGFR and uACR laboratory results that are often shared with patients through patient portals exclusively use medical terminology. Paradoxically, educational tools that do not use similar medical vocabulary may thus complicate discussions about kidney disease due to disparate terminology.

Prior investigations that assess CKD awareness using lay terms such as “kidney problem” have shown incremental improvement but overall residual low awareness [17]. In an effort to increase this awareness and in response to usability testing, NKF decided to keep the eGFR and uACR terms in the Kidney Score Platform self-assessment tool, recognizing that many patients would not know the names of these diagnostic tests. The terms were introduced with simple descriptive phrases—for example *“*albumin-creatinine ratio (uACR) or a type of protein in the urine”—to share the medical terminology that is used by clinical laboratories and national awareness campaigns while promoting discussions between patients and their clinicians about the usefulness and importance of these diagnostic tests to identify risks of CKD. Testing these changes in a real-world environment will demonstrate whether this strategy outweighs the risks of alienating individuals with medical terminology, particularly those who may be in denial about their chronic health conditions that increase the risk of developing CKD.

Connecting medical and lay terms may also require examples or approaches to enhance understanding. Conceptually, describing medical terminology like eGFR in ml/min/1.73m^2^ as a percentage of kidney function, as suggested by this investigation, may help patients understand the relationship between medical and lay terms. Using the test percent performance concept for percent of kidney function with 60 or higher being normal may also make conceptual sense to patients, as 60% is approximately the customary level for scholarly examination failure in the United States. In addition, some patients may better conceptualize eGFR and uACR with the images that were explored in this investigation, with heat map colors (from low risk) of green, yellow, orange, and (high risk) red. Study findings are hypothesis generating for future investigations regarding methods to connect medical and lay terminology as well as integration of images to illustrate the relationships.

The second key theme identified the importance of linking education about kidney disease with action-oriented recommendations that can decrease the risk of kidney disease or CKD progression. Clinicians and public health officials may consider awareness of kidney disease an important outcome on its own. However, patients may consider awareness of chronic conditions only as a means to an end—a transitory step that may not lead to improved health unless the education is coupled with actionable risk-reduction tools and motivational interviewing [22]. Empowering patients with concrete examples of self-care activities that they can discuss with their clinician may help overcome the negative association between CKD awareness, poor control of CKD risk factors, and adverse health outcomes [23,24]. In direct response to this usability study, the NKF revised the “Results” page of the Kidney Score Platform. This page now has streamlined verbiage explaining the recommended tests to detect CKD, language that encourages users to read about their risk factors for kidney disease even if their underlying conditions are under control, more visible links for patients to read about actions they can take to reduce their risk of kidney disease, and additional links related to kidney-friendly diets and healthy lifestyle choices.

Both content and sequence are important elements of CKD educational media design. Workflow for time-constrained primary care clinicians is a major barrier to clear and effective bidirectional communication about kidney health, kidney disease risk, and the interplay with an array of cardiometabolic risk conditions. We designed the study to occur before the veteran-practitioner encounters to address this barrier that in turn contributed to the clinicians recommending that the Kidney Score Platform education be delivered before the visits. Additionally, the platform could also be used after a clinician visit, allowing a modified education tailored to the interventions emphasized in the encounter or the after-visit summary for reinforcement.

### Limitations

The results of this study should be taken in context of its limitations. Although our sample size was larger than the recommended range of 5 to 7 participants for usability testing [25], our sample size was relatively small, which limits the inferences we can draw from our findings. Participants were recruited from 2 Veterans Administration clinics, which may not be representative of primary care providers or the general adult population at risk for kidney disease nor those who deliver or receive care in nonintegrated health care delivery systems. Furthermore, all veterans were native English speakers; results cannot be generalized to populations with limited English proficiency at risk for kidney disease, for whom more targeted tools and interventions may be needed to bridge the communication divide about kidney disease.

### Conclusions

Information derived from this usability study enabled the NKF to strengthen the Kidney Score Platform tool to promote its usefulness as an empowering adjunct to care and provided some key themes that will be applicable to the development of future educational materials.

## Abbreviations

CKD: chronic kidney disease eGFR: estimated glomerular filtration rate
NKF: National Kidney Foundation
uACR: urine albumin-creatinine ratio
VA-NYHHS: VA NY Harbor Healthcare System
VA-CTHS: VA CT Healthcare System at West Haven

## References

[1] Go AS, Chertow GM, Fan D, McCulloch CE, Hsu C (2004). Chronic kidney disease and the risks of death, cardiovascular events, and hospitalization. N Engl J Med.

[2] Bodenheimer T, Lorig Kate, Holman Halsted, Grumbach Kevin (2002). Patient self-management of chronic disease in primary care. JAMA.

[3] Chu CD, McCulloch CE, Banerjee T, Pavkov ME, Burrows NR, Gillespie BW, Saran R, Shlipak MG, Powe NR, Tuot DS, Centers for Disease ControlPrevention Chronic Kidney Disease Surveillance Team (2020). CKD awareness among US Adults by future risk of kidney failure. Am J Kidney Dis.

[4] Tuot DS, Plantinga LC, Hsu C, Jordan R, Burrows NR, Hedgeman E, Yee J, Saran R, Powe NR (2011). Chronic kidney disease awareness among individuals with clinical markers of kidney dysfunction. CJASN.

[5] Chao C, Lee Y, Yang K, Peng J, Li C, Chen S, Han D, Huang J, Cogent Study Group (2018). Impact of self-report and eGFR-based chronic kidney disease on the risk of chronic kidney disease-related complications and geriatric syndromes in community-dwelling older adults. Kidney Blood Press Res.

[6] Greer R, Crews Deidra C, Boulware L Ebony (2012). Challenges perceived by primary care providers to educating patients about chronic kidney disease. J Ren Care.

[7] Shin Jung-Im, Chang Alex R, Grams Morgan E, Coresh Josef, Ballew Shoshana H, Surapaneni Aditya, Matsushita Kunihiro, Bilo Henk J G, Carrero Juan J, Chodick Gabriel, Daratha Kenn B, Jassal Simerjot K, Nadkarni Girish N, Nelson Robert G, Nowak Christoph, Stempniewicz Nikita, Sumida Keiichi, Traynor Jamie P, Woodward Mark, Sang Yingying, Gansevoort Ron T, Prognosis Consortium CKD (2021). Albuminuria testing in hypertension and diabetes: an individual-participant data meta-analysis in a global consortium. Hypertension.

[8] Lee J, Chu C, Guzman D, Fontil V, Velasquez A, Powe N, Tuot D (2019). Albuminuria testing by race and ethnicity among patients with hypertension with and without diabetes. Am J Nephrol.

[9] Greer RC, Cooper LA, Crews DC, Powe NR, Boulware LE (2011). Quality of patient-physician discussions about CKD in primary care: a cross-sectional study. Am J Kidney Dis.

[10] Sperati CJ, Soman S, Agrawal V, Liu Y, Abdel-Kader K, Diamantidis CJ, Estrella MM, Cavanaugh K, Plantinga L, Schell J, Simon J, Vassalotti JA, Choi MJ, Jaar BG, Greer RC (2019). Primary care physicians’ perceptions of barriers and facilitators to management of chronic kidney disease: a mixed methods study. PLoS ONE.

[11] van Dulmen S, Roodbeen R, Schulze L, Prantl K, Rookmaaker M, van Jaarsveld B, Noordman J, Abrahams A (2022). Practices and perspectives of patients and healthcare professionals on shared decision-making in nephrology. BMC Nephrol.

[12] Boulware LE, Carson KA, Troll MU, Powe NR, Cooper LA (2009). Perceived susceptibility to chronic kidney disease among high-risk patients seen in primary care practices. J Gen Intern Med.

[13] Tuot DS, Crowley ST, Katz LA, Leung J, Alcantara-Cadillo DK, Ruser C, Talbot-Montgomery E, Vassalotti JA (2020). The Kidney Score pPlatform for patient and clinician awareness, communication, and management of kidney disease: protocol for a mixed methods stud. JMIR Res Protoc.

[14] Vassalotti JA, Boucree SC (2022). Integrating CKD into US primary care: bridging the knowledge and implementation gaps. Kidney Int Rep.

[15] Maramba I, Chatterjee A, Newman C (2019). Methods of usability testing in the development of eHealth applications: A scoping review. Int J Med Inform.

[16] Levey A, Eckardt K, Dorman N, Christiansen S, Cheung M, Jadoul M, Winkelmayer W (2020). Nomenclature for kidney function and disease: report of a Kidney Disease: Improving Global Outcomes (KDIGO) Consensus Conference. Kidney Dis (Basel).

[17] Tuot DS, Zhu Y, Velasquez A, Espinoza J, Mendez CD, Banerjee T, Hsu C, Powe NR (2016). Variation in patients’ awareness of CKD according to how they are asked. CJASN.

[18] Tuot D, Wong Karen K, Velasquez Alexandra, Crews Deidra C, Zonderman Alan B, Evans Michele K, Powe Neil R (2019). CKD awareness in the general population: performance of CKD-specific questions. Kidney Med.

[19] Chu CD, Chen MH, McCulloch CE, Powe NR, Estrella MM, Shlipak MG, Tuot DS (2021). Patient awareness of CKD: a systematic review and meta-analysis of patient-oriented questions and study setting. Kidney Med.

[20] Tuot DS, Davis E, Velasquez A, Banerjee T, Powe NR (2013). Assessment of printed patient-educational materials for chronic kidney disease. Am J Nephrol.

[21] Palazzuoli A, Tecson KM, Ronco C, McCullough PA (2021). Nomenclature for kidney function from KDIGO: shortcomings of terminology oversimplification. Cardiorenal Med.

[22] Loskutova N, Tsai Adam G, Callen Elisabeth, Ajayi Kemi, Carroll Jennifer K, Harrington Michael, Turner Tamela J, Pace Wilson D (2018). Differences in perspectives regarding diabetes management between health care providers and patients. Transl Behav Med.

[23] Tuot DS, Plantinga LC, Judd SE, Muntner P, Hsu C, Warnock DG, Gutiérrez Orlando M, Safford M, Powe NR, McClellan WM, REGARDS Investigators (2013). Healthy behaviors, risk factor control and awareness of chronic kidney disease. Am J Nephrol.

[24] Tummalapalli S, Vittinghoff E, Crews D, Cushman M, Gutiérrez Orlando M, Judd S, Kramer H, Peralta C, Tuot D, Shlipak M, Estrella M (2020). Chronic kidney disease awareness and longitudinal health outcomes: results from the reasons for geographic and racial differences in stroke study. Am J Nephrol.

[25] Faulkner L (2003). Beyond the five-user assumption: benefits of increased sample sizes in usability testing. Behav Res Methods Instrum Comput.

